# Multiple Bilateral Cystoma of the “Organ of Giraldes”*Reported o the Louisville, Clinical Srciety.

**Published:** 1897-04

**Authors:** William C. Dugan

**Affiliations:** Professor of the Principles and Practice of Surgery and Surgical Pathology in the Louisville Medical College, etc., Louisville, Ky.


### MULTIPLE, BILATERAL CYSTOMA OF THE "ORGAN OF GIRALDES."*

By WILLIAM C. DUGAN, M. D.

Professor of the Principles and Practice of Surgery and Surgical Pathology in the Louisville Medical College, etc., Louisville, Ky.

The specimen, which I present for your examination, was removed from the left side of the scrotum of a patient aged forty-eight years; the tumor removed from the left epididymis was very much larger than this, made up of four or five cysts; both of them were dissected out without rupture, but my assistant, in handling the larger tumor, accidently ruptur-

*Reported [illegible] the Louisville, Clinical Srciety.

ed it, which I regret very much, as I had hoped to present both of them in reporting the case.

These tumors are very peculiar. I have seen several such cases. The first one met with was as large as a cocoanut, and came on, according to the statement of the patient, in a few hours, and was thought to be a hernia. The patient had all the symptoms of a hernia, vomiting, pain, etc. In an examination of this case I recognized the condition, and operated upon the patient, finding as I had suspected, a large cyst springing from the lower part of the epididymis, the testicle was pushed downwards and forward, and by pulling the skin taut, I could see distinctly the grooved condition separating the tumor from the testicle below. I made the usual incision like that for the cure of hernia, and then by a careful dissection, lifting off layer after layer—after apparently getting down to the tumor proper I am sure we removed half a dozen layers—finally dissected the tumor loose without opening the the tunica vaginalis, and found that it sprang from the back part of the epididymis, from what is known as "the organ of Giraldes," which bears a good deal of resemblance to the organ of Rosenmuller in the other sex. These tumors are the analogue of the parovarian cyst as I understand it. I am sure they are not infrequently diagnosed encysted hydrocele of the cord or of the testes. I witnessed an operation recently by one of our leading surgeons, in which he made no attempt to enucleate the tumor, but opened it and stitched it to the skin. I confess I made the same mistake once.

REMARKS.

DR. W. H. WATHEN:—"We do not see many specimens of this sort, especially as beautiful as the ones presented. These are from cœcal tubules of remnants of the Wolffian duct, which is in the paradidymis of the male, and they are morphologically analogous to small parovarian cysts found in the broad ligament of the female; and if you will submit the walls of these cysts to microscopical examination, you will find the histological conditions that exist in small parovarian cysts.

"The pathology of these tumors can not be understood unless we understand the embryology of the male and female generative organs. It is then very easily understood, and the treatment is simple, and easily carried out, and of course universally successful. There is no danger in these tumors except that they are disagreeable because of their size, and the pressure upon adjacent structures. I dare say that Dr. Dugan will have no trouble, and that there will be a speedy and perfect recovery in this case.

